# Features of Mammalian microRNA Promoters Emerge from Polymerase II Chromatin Immunoprecipitation Data

**DOI:** 10.1371/journal.pone.0005279

**Published:** 2009-04-23

**Authors:** David L. Corcoran, Kusum V. Pandit, Ben Gordon, Arindam Bhattacharjee, Naftali Kaminski, Panayiotis V. Benos

**Affiliations:** 1 Department of Human Genetics, Graduate School of Public Health, University of Pittsburgh, Pittsburgh, Pennsylvania, United States of America; 2 Dorothy P. and Richard P. Simmons Center for Interstitial Lung Disease, Division of Pulmonary, Allergy and Critical care Medicine, University of Pittsburgh School of Medicine, Pittsburgh, Pennsylvania, United States of America; 3 Genomics, Agilent Technologies, Inc., Santa Clara, California, United States of America; 4 Department of Computational Biology, University of Pittsburgh School of Medicine, Pittsburg, Pennsylvania, United States of America; 5 Department of Biomedical Informatics, University of Pittsburgh School of Medicine, Pittsburg, Pennsylvania, United States of America; Lawrence Berkeley National Laboratory, United States of America

## Abstract

**Background:**

MicroRNAs (miRNAs) are short, non-coding RNA regulators of protein coding genes. miRNAs play a very important role in diverse biological processes and various diseases. Many algorithms are able to predict miRNA genes and their targets, but their transcription regulation is still under investigation. It is generally believed that intragenic miRNAs (located in introns or exons of protein coding genes) are co-transcribed with their host genes and most intergenic miRNAs transcribed from their own RNA polymerase II (Pol II) promoter. However, the length of the primary transcripts and promoter organization is currently unknown.

**Methodology:**

We performed Pol II chromatin immunoprecipitation (ChIP)-chip using a custom array surrounding regions of known miRNA genes. To identify the true core transcription start sites of the miRNA genes we developed a new tool (CPPP). We showed that miRNA genes can be transcribed from promoters located several kilobases away and that their promoters share the same general features as those of protein coding genes. Finally, we found evidence that as many as 26% of the intragenic miRNAs may be transcribed from their own unique promoters.

**Conclusion:**

miRNA promoters have similar features to those of protein coding genes, but miRNA transcript organization is more complex.

## Introduction

MicroRNAs (miRNAs) are short, ∼22 nt, single-stranded RNAs that act as regulators of genes' expression. By virtue of base complementarity, they bind to their target gene mRNAs and can block translation or accelerate their degradation [1]. miRNAs have been implicated in a variety of human diseases [2], [3] and more recent studies showed their association with particular cellular pathways [4].

Although miRNA genes play an important role in many biological processes, little is known about their transcriptional regulation. Currently, it is believed that most miRNA genes are transcribed by RNA polymerase II (Pol II) [5], [6], although some exceptions exist [7]. A first step toward understanding miRNA regulation is to identify their transcription start sites (TSSs). Currently, only a small number of human miRNA genes have confirmed TSSs [5], [8], [9], which is insufficient for studying the promoter sequence features and for comparison with protein coding genes. Due to this lack of information, all studies attempting to analyze the miRNA core promoters have focused on the area immediately upstream of the computational prediction of the pri-miRNA [10], [11], [12]. While these regions exhibit similar conservation patterns to the promoters of protein coding genes [13] their potential to act as promoters is still unknown. Identifying the active core miRNA promoters will thus allow us to study particular pri-miRNA characteristics such as transcript length and core promoter features. Recently, two studies that utilized high-throughput genomic techniques offered a first glimpse into the likely location and sequence characteristics of human miRNA TSSs [14], [15]. In addition, two other studies involving high-throughput data from mouse and *C. elegans* offered insights on miRNA gene transcription in these species [14], [16].

Existing algorithms for modeling Pol II core promoters vary both in methodology and in performance. Previous algorithms have used transcription factor binding site frequencies [17], the size and location of CpG islands [18], and the physical properties of the DNA. Algorithmically, neural networks [19], relevance vector machines [20], and additive logistic regression with boosting have been applied [21].

To better understand the transcriptional regulation of miRNAs we performed chromatin immunoprecipitation (ChIP)-chip for the Pol II complex using a custom designed miRNA location array. After comparing different DNA features, we developed an efficient Support Vector Machine (SVM) based method for Pol II core promoter classification (*Core Promoter Prediction Program*, CPPP). We applied these tools to identify miRNA TSSs, better understand how intergenic and intragenic (*i.e.*, intronic or exonic) miRNA genes are transcribed and to compare the features of their promoters with those of the protein coding genes.

## Results and Discussion

### Identification of regions containing pri-miRNA TSSs from Pol II ChIP-chip data

**Figure 1:**
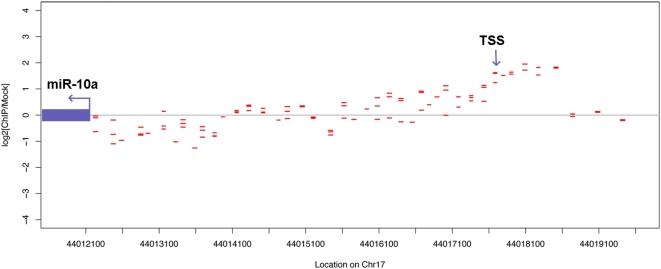
Pol II ChIP-chip results for miR-10a. The blue arrow represents the location and transcriptional direction of hsa-miR-10a. The red dashes represent the location and value of the ChIP-chip probes. *TSS* – transcription start site of this miRNA.

To identify the TSS for pri-miRNAs, ChIP-chip was performed on A549 lung epithelial cells with a Pol II-specific antibody, as described in *Materials and Methods*. Statistical analysis [22] was used to identify windows of 1 Kb in length that exhibit significant Pol II signals (immunoprecipitated DNA *vs.* background). The nearest statistically significant window to the 5′ end of each of the 531 known pre-miRNAs was further analyzed with our algorithm to predict whether it contained the miRNA TSS. The custom-made tiling array we used included 50 Kb upstream of each known miRNA gene (see *Materials and Methods*). This distance threshold is consistent with previous studies that showed high correlation of expression between miRNA genes located up to 50 Kb apart [23]. Our method resulted in 34 intergenic pre-miRNAs or polycistronic pri-miRNAs having a statistically significant Pol II signal associated with them (**Table 1**). Regions with a significant Pol II signal that also overlapped the 5′ end of a known gene (as identified by the UCSC table browser [24], [25]) were excluded from subsequent analysis. This was necessary because the ChIP-chip data cannot distinguish shared core promoter regions. An example of the distribution of the Pol II binding signals and the identified TSS of the pri-miR-10a is presented in **Figure 1**.

**Table 1:** Identification of promoters of intergenic miRNA genes.

miRNA	Chromosomal location	ChIP-chip region	CPPP Model	Predicted TSS (CPPP)	Distance
miR-200b∼miR-200a∼miR-429	Chr1: 1092346 (+)	[1082033, 1083782]	CpG+	1083333	8763
miR-34a	Chr1: 9134423 (−)	[9162283, 9166532]	CpG+	9163733	29310
miR-101-1	Chr1: 65296779 (−)	[65304283, 65307532]	CpG+	65305833	9054
miR-181a-1∼miR-181b-1	Chr1: 197094905 (−)	[197125783, 197127032]	**CpG−**	*none predicted*	***NA***
miR-202	Chr10: 134911115 (−)	[134919994, 134925743]	CpG+	134924844	8879
miR-210	Chr11: 558198 (−)	[559355, 560354]	CpG+	*none predicted*	***NA***
miR-194-2∼miR-192	Chr11: 64415487 (−)	[64416605, 64418104]	CpG−	64416930	1193
miR-200c∼miR-141	Chr12: 6943122 (+)	[6940546, 6942545]	CpG+	6941146	1976
let-7i	Chr12: 61283732 (+)	[61279796, 61291045]	CpG+	61283796	506
miR-379∼miR411∼…∼miR-410∼miR-656	Chr14: 100558155 (+)	[100524119, 100525868]	**CpG−**	*none predicted*	***NA***
miR-193b	Chr16: 14305324 (+)	[14302031, 14310280]	CpG+	14304581	743
miR-138-2	Chr16: 55449930 (+)	[55439531, 55441030]	CpG−	55439856	9824
miR-497∼miR-195	Chr17: 6862065 (−)	[6863309, 6865058]	CpG−	6864759	2444
miR-10a	Chr17: 44012308 (−)	[44017059, 44018808]	CpG+	44017709	5401
miR-196a-1	Chr17: 44064920 (−)	[44078809, 44080558]	CpG+	44079509	14589
**miR-21**	**Chr17: 55273408 (+)**	**[55267309, 55276558]**	**CpG−**	**55271984**	**1174**
miR-122	Chr18: 54269285 (+)	[54235566, 54236565]	CpG−	54235891	33144
**miR-23a∼miR-27a∼miR-24-2**	**Chr19: 13808473 (**−**)**	**[13807348, 13809097]**	**CpG−**	**13808448**	**0**
miR-181c∼miR-181d	Chr19: 13846512 (+)	[13832848, 13834847]	**CpG−**	*none predicted*	***NA***
miR-99b∼let-7e∼miR125a	Chr19: 56887676 (+)	[56882098, 56886347]	CpG+	*none predicted*	***NA***
miR-216a∼miR-217	Chr2: 56069698 (−)	[56072783, 56074282]	CpG−	56073933	3985
miR-301b∼miR-130b	Chr22: 20337269 (+)	[20335283, 20337282]	CpG+	20336583	686
let-7a-3∼let-7b	Chr22: 44887292 (+)	[44879283, 44883032]	CpG+	44881933	5109
miR-206∼miR-133b	Chr6: 52117105 (+)	[52096878, 52098877]	CpG−	52098453	18402
miR-30a	Chr6: 72170045 (−)	[72164628, 72176377]	CpG−	72174203	3908
miR-129-1	Chr7: 127635160 (+)	[127593752, 127595501]	CpG+	127594092	41068
miR-183∼miR-96∼miR-182	Chr7: 129202090 (−)	[129206752, 129207751]	CpG+	129207202	5112
miR-29b-1∼miR-29a	Chr7: 130212838 (−)	[130219002, 130223501]	CpG−	130223027	9939
miR-30d∼miR-30b	Chr8: 135886370 (−)	[135913283, 135915782]	CpG+	135914133	27763
let-7a-1∼let-7f-1∼let-7d	Chr9: 95978059 (+)	[95966631, 95971380]	CpG+	95969131	9928
miR-181a-2∼miR-181b-2	Chr9: 126494541 (+)	[126459631, 126464380]	CpG−	126460831	33460
miR-222∼miR-221	ChrX: 45491474 (−)	[45504862, 45507861]	CpG−	45506782	15308
miR-542∼miR-450a-2∼miR-450a-1∼miR-450b	ChrX: 133503133 (−)	[133502362, 133506611]	CpG+	133505762	2629
miR-505	ChrX: 138834056 (−)	[138842362, 138844111]	CpG+	138843122	9066

*miRNA*: miRNA gene symbol, multiple symbols designate cluster of co-expressed miRNAs; *Chromosomal location*: the chromosomal position and orientation of the miRNA gene; *ChIP-chip region*: the nearest region with a statistically significant peak; *CPPP model*: the CpG (CpG+) or non-CpG (CpG−) model used for the TSS prediction; *Predicted TSS*: TSS predicted by CPPP; *Distance*: the distance of the predicted TSS from the most 5′ pre-miRNA transcript. Bold letters designate previously verified TSSs.

The miR-23a∼miR-27a∼miR24-2 cluster is probably the best-studied human intergenic pri-miRNA transcript. Lee *et al.* [5] have shown that the TSS for this cluster is located 124 nucleotides upstream of miR-23a, which our ChIP-chip data analysis confirmed. The ChIP-chip data was also able to confirm the previously reported pri-miRNA TSS listed by Fujita and Iba [26] for miR-21 (**Table 1**). The distance between the Pol II peaks and the location of the pre-miRNA varies substantially between genes, from a minimum of zero to a maximum of 40 Kb. The average and median values are 10.8 Kb and 8.7 Kb, respectively.

The analysis of the Pol II ChIP-chip data also provided insights into how intragenic (intronic, exonic) miRNAs are transcribed. Currently, it is believed that they are transcribed along with their host gene [6]. Indeed, for many intragenic miRNAs the nearest significant upstream Pol II ChIP-chip peak region overlapped the 5′ region of their host gene (**Table 2**). These cases include a few miRNAs that were previously shown to be co-transcribed with their host gene, such as miR-146a [27] and the miR-17∼miR-18a∼miR-19a∼miR-20a∼miR-19b-1∼miR-92a-1 cluster [9]. Interestingly, the analysis found that some of the intragenic miRNA genes may be transcribed by their own promoter (**Table 3**), which was also observed in the recent analysis by Ozsolak *et al.* [15]. We note that in contrast with the promoters of intergenic miRNA genes, CpG islands were identified only in 3 of the 11 intragenic promoters. The distance between the Pol II peak and the beginning of the (intragenic) pre-miRNA gene also varies between zero and 41 Kb, but with a higher number of TSSs observed at longer distances (average and median = 19 Kb).

**Table 2:** Intragenic miRNAs who's nearest ChIP-chip peak overlaps the host gene's TSS.

miRNA	Host Gene	Chromosomal location	ChIP-chip region
miR-30e∼miR30c-1	NFYC	Chr1: 40992613 (+)	[40946783, 40950532]
miR-186	ZRANB2	Chr1: 71305987 (−)	[71316783, 71320532]
miR-130a	AK096335	Chr11: 57165246 (+)	[57161605, 57163604]
miR-148b	COPZ1	Chr12 53017266 (+)	[53004046, 53006295]
miR-26a-2	CTDSP2	Chr12: 56504742 (−)	[56524546, 56528295]
miR-15a∼miR-16-1	DLEU2	Chr13: 49521338 (−)	[49551648, 49555397]
**miR-17∼miR-18a∼miR-19a∼miR-20a∼miR-19b-1∼miR-92a-1**	**C13orf25 v_1**	**Chr13: 90800859 (+)**	**[90798648, 90800647]**
miR-423	CCDC55	Chr17: 25468222 (+)	[25467059, 25470058]
miR-301a∼miR-454	FAM33A	Chr17: 54583364 (−)	[54583809, 54589308]
miR-330	EML2	Chr19: 50834185 (−)	[50833598, 50834597]
miR-26b	CTDSP1	Chr2: 218975612 (+)	[218968033, 218974282]
miR-103-2	PANK2	Chr20: 3846140 (+)	[3816001, 3820000]
miR-185	C22orf25	Chr22: 18400661 (+)	[18387533, 18389782]
miR-191∼miR-425	DALRD3	Chr3: 49033146 (−)	[49026104, 49038353]
miR-15b∼miR-16-2	SMC4	Chr3: 161605069 (+)	[161598354, 161603353]
miR-378	PPARGC1B	Chr5: 149092580 (+)	[149089935, 149091684]
miR-103-1	PANK3	Chr5: 167920556 (−)	[167938685, 167940184]
miR-335	MEST	Chr7: 129923187 (+)	[129912502, 129914001]
miR-31	LOC554202	Chr9: 21502184 (−)	[21539381, 21557130]
miR-421	AK125301	ChrX: 73355021 (−)	[73377862, 73379611]
miR-374b∼miR-374a∼miR-545	AK057701	ChrX: 73355178 (−)	[73421362, 73431611]
miR-361	CHM	ChrX: 85045368 (−)	[85188362, 85189861]
miR-503	MGC16121	ChrX: 133508094 (−)	[133506612, 133515611]
miR-452∼miR-224	GABRE	ChrX: 150878840 (−)	[150889112, 150894611]
miR-22	MGC14376	Chr17: 1564031 (−)	[1563059, 1569558]
miR-636	SFRS2	Chr17: 72244225 (−)	[72244059, 72246308]
**miR-146a**	**DQ658414**	**Chr5: 159844936 (+)**	**[159826435, 159828934]**

*Host gene*: the gene whose intron the miRNA was found in. Other column names as in Table 1. Bold letters designate genes that are known to be co-transcribed with their host genes.

**Table 3:** Identification of promoters for *intragenic* miRNA genes.

miRNA	Host Gene	Chromosomal location	ChIP-chip region	CPPP Model	Predicted TSS (CPPP)	Distance
miR-107	PANK1	Chr10: 91342564 (−)	[91382494, 91383493]	CpG−	91382844	40030
let-7a-2∼miR-100	AK091713	Chr11: 121522511 (−)	[121521855, 121523854]	***NA***	*none predicted*	***NA***
miR-190	TLN2	Chr15: 60903208 (+)	[60860703, 60861952]	CpG−	60861428	41530
miR-99a∼let-7c	C21orf34	Chr21: 16833279 (+)	[16826951, 16832700]	CpG−	16827826	5203
miR-125b-2	C21orf34	Chr21: 16884427 (+)	[16880451, 16883950]	CpG−	16880951	3226
miR-26a-1	CTDSPL	Chr3: 37985898 (+)	[37961854, 37963353]	CpG−	37962529	23119
miR-196b	HOXA9	Chr7: 27175707 (−)	[27178752, 27180251]	CpG+	27178802	3095
miR-489∼miR-653	CALCR	Chr7: 92951267 (−)	[92953002, 92954251]	***NA***	*none predicted*	***NA***
miR-101-2	RCL1	Chr9: 4840296 (+)	[4827381, 4828630]	CpG−	4828281	11765
miR-491	KIAA1797	Chr9: 20706103 (+)	[20673131, 20677880]	CpG+	20677181	28922
miR-204	TRPM3	Chr9: 72614820 (−)	[72633881, 72634880]	***NA***	*none predicted*	***NA***
miR-7-1	HNRPK	Chr9: 85774592 (−)	[85774131, 85775630]	CpG−	85775081	239
mir-23b∼miR-27b∼miR-24-1	C9orf3	Chr9: 96887310 (+)	[96846381, 96860880]	CpG+	96855881	31429
**miR-32**	**C9orf5**	**Chr9: 1108483999 (−)**	**[110866881, 110868380]**	**CpG−**	**110867881**	**19232**
miR-448	HTR2C	ChrX: 113964272 (+)	[113955612, 113956861]	***NA***	*none predicted*	***NA***

Column names as in Table 1 and 2. Bold letters designate genes whose expression was found to be anti-correlated with their host genes.

### Modeling Pol II core promoter features with *n*-mers and weight matrices

In the following section, we describe the development of CPPP, a novel SVM-based method for prediction of Pol II TSSs. CPPP was used for the identification of the miRNA TSSs from the ChIP-chip data and for comparing the features of the miRNA promoters to those of protein coding gene promoters.

It is known that the genomic regions immediately upstream of the TSS of protein coding genes exhibit high levels of sequence conservation [13], [28], [29], [30], [31], which is probably related to the high concentration of *cis*-regulatory sites in this region [32]. All of the existing algorithms for modeling Pol II core promoters have used this property to different extents. Generally one can model DNA target sites using either *n*-mer frequencies or weight matrices, commonly known as position-specific scoring matrices (PSSM) [33]. The first class of methods (also termed *enumerating* or *dictionary-based* methods; e.g., [34], [35], [36], [37]) is better suited for representation of the binding preferences of those transcription factors that have a restricted set of DNA targets. *n*-mer frequencies have been used in the past to model Pol II core promoters either alone [12] or in conjunction with some promoter entropy measure [38]. However, the DNA targets of most transcription factors are not highly conserved, which is the reason why PSSM models are widely used for representing DNA motifs. Regardless, using PSSMs for Pol II core promoter recognition has also its limitations. First, the currently known DNA motifs are redundant, not only because the available databases contain multiple matrices for the same factor, but also because structurally similar transcription factors are known to recognize similar “core” motifs [29], [39]. Second, the binding preferences are known only for a small percent of the transcription factor proteins and protein complexes. For example, TRANSFAC database [40] currently has annotated 2,113 mammalian transcription factors, but it only contains 601 binding models. Third, even if the binding preference of a given transcription factor is known, the task of determining whether it binds to a given promoter is not trivial, mainly due to the high false positive prediction rate [41], [42]. Despite the above limitations, PSSM models have been used extensively in the past for Pol II core promoter identification [10], [21], [43].

The problems of PSSM model redundancy and the relatively small number of transcription factors with known binding preferences can be diminished if one uses familial binding profiles (FBPs) [39]. FBPs represent an “average” of the binding preferences of related transcription factors. They are based on the fact that transcription factors of the same structural group typically bind to similar sets of sequences. This method not only reduces the PSSM model redundancy, but also offers models for the transcription factors for which the binding preference is currently unknown (since the transcription factors with unknown preferences are likely to belong in one of the existing families). Sandelin and Wasserman initially built a set of 11 FBPs using a semi-manual method [44]. In that study, the zinc finger proteins were excluded from the FBP construction due to their high degree of target promiscuity, which in turn makes them difficult to cluster correctly. More recently, Mahony *et al.* [29] used an automatic method to construct 17 FBPs. This set of FBPs includes all but the C2H2 the zinc finger (sub)family.

Using the same clustering method developed in Mahony *et al.* [29] we built 31 new FBPs from the C2H2 zinc finger proteins. These 31 new FBPs together with the 17 FBPs from the Mahony *et al.* study were used in the subsequent analysis.

### Evaluating Core Promoter Features Using Support Vector Machines

In order to better understand how various features contribute to the characterization of the Pol II core promoters we compared them using an SVM [45], [46]. The SVM methodology was chosen because it can combine multiple types of evidence (features) under the same general framework. In this study, we used (a) the *n*-mer frequencies (*n = 3,4*) and (b) matches to the set of 48 generalized DNA binding profiles (FBPs) as features of the SVM, and (c) the GC content. The reason for using GC content as an additional feature in the SVM training is that it seems to be a prominent feature in a subset of eukaryotic promoters [47].

**Figure 2:**
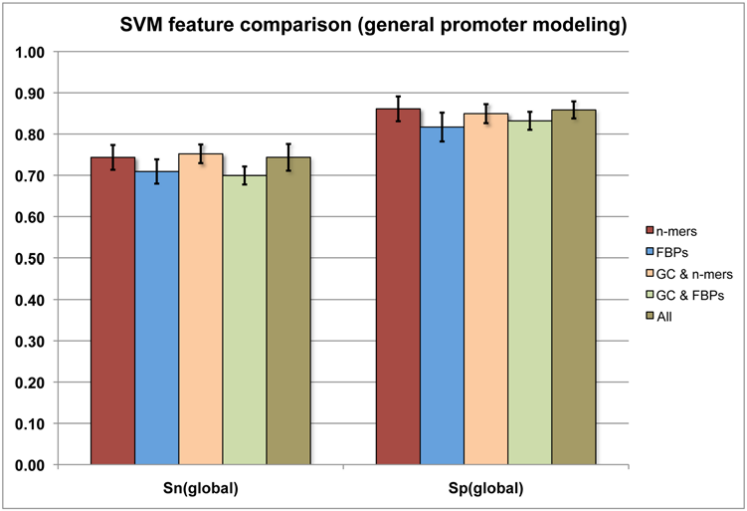
Performance of the *n*-mers and FBPs (alone and in combination) in predicting Pol II core promoter regions. *Sn* – sensitivity, *Sp* – specificity.

Overall, we constructed and compared five SVM models: **(1)** FBPs only (48 features), **(2)** *n*-mers only (*n = 3,4*) (320 features), **(3)** FBPs+GC content, **(4)** *n*-mers+GC content, and **(5)** FBPs+*n*-mers+GC content. All models were trained on the same set of 3,015 verified core promoters of protein coding genes (positive examples; see *Materials & Methods*) and 3,015 randomly chosen intergenic sequences (negative examples; see *Materials and Methods*). Performance was measured by a 20× cross-validation in which 75% of the examples in each dataset were used for training and the remaining 25% for testing. The results are presented in **Figure 2**, and indicate that the *n*-mer-based models perform generally better than the FBP-based models, both in terms of sensitivity (percent of correctly predicted positive examples) and specificity (percent of true positive examples among all predictions). For example, the “*n*-mer only” SVM model (*n* = *3*, *4*) exhibited *S_N_ = 74.3%* and *S_P_ = 86.1%* compared to *S_N_ = 70.8%* and *S_P_ = 82.2%* of the “FBP only” model. It should be noted, however, that *none of these differences is statistically significant*. Based on these results, one may choose to use FBPs for this type of modeling, especially in species where the number of training examples is limited.

The SVM results reported above were based on the dot plot kernel function (linear discriminator). Tests with polynomial (3^rd^ order) and radial kernels gave the same or slightly worse results (*data not shown*). Also, all SVM models were constructed using random intergenic regions as background (see *Materials and Methods*) instead of the intronic regions previous studies have used [38]. Evaluation with intronic sequences as background was found to be slightly worse (*data not shown*).

We note that other studies have occasionally reported better performance (*e.g.*, [12], [38]). We believe this is due to the smaller size of the datasets they used and the type of promoters these datasets contained. For example, Gangal and Sharma [38] reported *S_N_*>*87%* and *S_P_>86%*, but the 800 promoter sequences in their dataset were all taken from EPD [48]. EPD is highly enriched in promoters containing CpG islands (about 83% of the total.) A very powerful separation hyperplane can be created using these GC-rich promoters as positive set and the, generally AT-rich, intronic sequences as negative set. However this model is expected to perform poorly on non-CpG island promoters, as we will show. In our case, only half of the promoters in the training/testing dataset contained CpG islands. When the EPD dataset is used for training/testing in this study, our results are similar (intronic background) or slightly better (intergenic background) to those reported in Gangal and Sharma [38]. Nevertheless, we found that partitioning the promoters to those containing CpG islands and those lacking CpG islands improves the results substantially (see below).

### The effect of the presence or absence of CpG islands in the prediction efficiency of Pol II core promoters

In general, the frequency of CG dinucleotides in vertebrate genomes is lower than expected by chance [49]. This is due to the frequent conversion of methylated-CG into TG [50]. However, often the promoters of vertebrate genes contain stretches with high frequency of CG dinucleotides (*CpG islands*) [51]. These regions are often defined as 200 nt or more with GC content greater than 50% [18]. Ioshikhes and Zhang [18] have previously used this feature to predict the CpG island containing promoters with high efficiency. For this reason, we tested the prediction efficiency of the “*n*-mer only” and “FBP only” SVM models in mammalian core promoters in the presence or absence of CpG islands. Focus was placed on these two models because they are simpler than the composite model and their performance in the general dataset is the same or slightly better than the other models (**Figure 2**).

**Figure 3:**
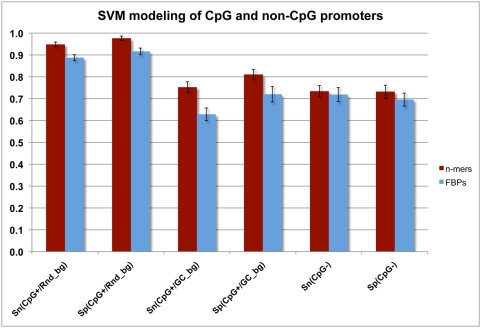
Performance of the SVM models in predicting CpG+ and CpG− promoters. Two SVM models were evaluated in the prediction of the CpG+ promoters: one with random intergenic background (CpG+/Rnd_bg) and one with intergenic background with similar GC content (CpG+/GC_bg). *Sn* – sensitivity, *Sp* – specificity.

The positive training set was partitioned into CpG containing promoters (CpG+) and non-CpG promoters (CpG−), for each of which a *n*-mer-based and a FBP-based SVM model were calculated. The negative dataset contained equal number of randomly selected sequences from the intergenic parts of the genome (see *Materials and Methods*). The results demonstrate that if SVMs are trained in this way, then the prediction efficiency differs significantly between the two types of promoters. In particular, the “*n*-mer model” trained on CpG+ promoters exhibits *S_N_* = 94.8% (SD = 1.1%) and *S_P_* = 97.6% (SD = 1%) in the cross-validation tests. By contrast, when trained on CpG− promoters the “*n*-mer model” performs significantly worse (*S_N_* = 73.4%, SD = 2.6% and *S_P_* = 73.2%, SD = 2.9%) (**Figure 3**). The results with the “FBP model” are similar for both the CpG+ and CpG− trained models (**Figure 3**). Also, the results show that in general *n*-mer models perform slightly better than the corresponding FBP models regardless of the training (CpG+ or CpG− datasets) (**Figure 3**). Furthermore, the results show that *n*-mer-based models trained on CpG+ promoters tend to predict extremely well the CpG promoters (*S_N_ = 94.8%*, *S_P_ = 97.6%*), which agrees with previous reports [18]. We have discovered that this better performance can be attributed to the GC content of these promoters (compared to the background), and this could be misleading. When intergenic sequences with similar GC content were used as negative dataset during training, the efficiency of the *n*-mer-based SVM on CpG+ promoters was reduced to values similar to the prediction of the CpG− promoters with the CpG− model (*S_N_* = 75.3% with SD = 2.4% and *S_P_* = 80.0% with SD = 2.0%). Since our main aim in this report is to discover important promoter features, not simply the features of the CpG islands, in the following analysis we use the seemingly less efficient models (i.e., *n*-mer SVMs trained on CpG+ *vs.* GC-normalized intergenic background and CpG− *vs.* random intergenic background).

The program ‘gist-fselect’ from the *Gist* package [45] was used to evaluate the significance of each of the features of core promoter regions (*t*-test metric *p*-value was used to determine significance) in CpG and non-CpG promoters. The top 20 features (ranked by the *Fisher* score of the package) are presented in **Table 4**. Interestingly, despite correcting for GC content, the most significant features for the CpG+ promoters were CG containing *n*-mers. Also of interest is the *n*-mer ‘CTG,’ which is present in the top 20 most significant features of both models.

**Table 4:** The top 20 most significant *n*-mers for each of the two models and the Fisher score as well as the −log10 of their *p*-value from *Gist* package (*t*-test metric).

non-CpG			CpG		
Feature	−log_10_(p-value)	Fisher Score	Feature	−log_10_(p-value)	Fisher Score
CCCT	29.7925	0.152704	GCG	26.8458	0.136008
AGGG	26.8574	0.136658	GGCG	23.3817	0.11732
GCCC	23.9996	0.12122	**CTG**	**21.5833**	**0.107693**
CCC	23.6638	0.119395	CGC	21.3434	0.106413
TGTA	23.7021	0.119389	CCTG	18.0213	0.0887804
CCCC	23.6248	0.119181	TCCG	17.9756	0.088539
AAT	23.4104	0.117827	GCGG	17.8046	0.0876364
GAAG	22.2979	0.111908	TCGC	15.998	0.0781331
AGC	21.1428	0.105734	CGA	14.4589	0.0700828
TAC	20.8754	0.104254	CTGG	14.448	0.0700258
ATT	19.5344	0.0971108	CAGG	14.0166	0.0677778
TAAT	19.1561	0.0950535	CTA	13.9502	0.067432
ATTA	19.1021	0.0947959	CGGA	13.7632	0.0664587
TACA	19.0021	0.0942557	GCGC	13.7331	0.0663022
GTA	18.6868	0.0925992	CGCC	13.2385	0.0637315
AATA	18.6051	0.092075	CAC	12.9486	0.0622268
GGG	18.0089	0.0890836	CAG	12.6467	0.0606617
CTGC	18.0034	0.0890473	CGG	12.3375	0.0590611
CAGC	16.8798	0.0831072	CGCG	12.3167	0.0589534
**CTG**	**16.3289**	**0.0801748**	TCG	12.2470	0.0585926

Bold letters indicate the *n*-mer that appears to be a significant feature in both the CpG+ and CpG− models.

### Comparison of core promoters for protein coding and miRNA genes with SVM models

The ChIP-chip data showed that 34 of the intergenic miRNA genes had significant Pol II signals less than 50 Kb away. The 3 Kb regions surrounding the windows with the most significant Pol II peak were collected and the presence or absence of CpG islands was determined using the same method as in Zhao *et al.* [21]. CpG islands were identified in about 55% of these promoters. Subsequently, the corresponding SVM model (trained on CpG+ or CpG− promoters of protein coding genes) was used across the significant ChIP-chip region to identify the top scoring 500 bp window that contains the predicted TSS. The CPPP algorithm identified a TSS in the upstream regions of 29 out of the 34 intergenic miRNA genes (**Table 1**). Each of the five intergenic miRNAs for which CPPP was unable to identify a core promoter contained a 500 bp region that scored just below the threshold cutoff for identifying a core promoter from a background sequence (*data not shown*).

The number of Pol II associated intergenic miRNA genes is not large enough to retrain the SVM models and calculate significant sequence features. However, we can test whether the most significant features in the promoters of the protein coding genes (**Table 4**) are also overrepresented in the miRNA promoters. Comparison of all *n*-mer frequencies of the CpG promoters of protein coding genes with those of the miRNA genes resulted in a statistically significant difference of 5 *n*-mers (‘CAC’, ‘GCAC’, ‘CGGT, ‘GTAC’, and ‘CTTA’; Wilcoxon signed-rank test; *p*-value<0.05 after Bonferroni correction). However, the only 4-mer in the list of the top 50 most important features for the model was ‘CAC’. For the non-CpG miRNA promoters, we found no features with a statistically different frequency when compared to that of the protein coding genes.

### Computational analysis of potential promoters of intragenic miRNA genes

Intragenic miRNA genes are generally believed to be co-transcribed with their host genes. Overall, we found significant Pol II peaks associated with 43 intragenic miRNA genes or gene clusters. In 27 cases, the Pol II peak overlapped the promoter of the host gene (**Table 2**), but in 15 cases the Pol II peak was located within the host gene (**Table 3**). We scanned the ChIP identified regions with internal Pol II peaks with the corresponding SVM model (CpG+ or CpG−) and we found that 11 of these 15 intragenic genes contained a highly likely TSS region (**Table 3**.) This result indicates that 26% or more of the intragenic genes may be transcribed from their own promoter. In agreement with this finding, the miR-32 gene was previously shown to have a negative correlation with its host gene, *C9orf5* [23]. This is an important and interesting finding about the transcriptional regulation of intragenic miRNAs, although further biochemical validation is required.

### Comparison with ChIP-seq data

Marson and colleagues [14] recently performed ChIP-seq experiments with four general transcription factors in human and mouse cells. They then combined their data with those from previous studies on epigenetic markers. Using a variety of features such as evolutionary conservation and distance of the peak from the known miRNA genes, they assigned an *ad hoc* score to each putative TSS. A positive score indicated that the TSS is likely to belong to the miRNA and a negative score indicated that the TSS likely belonged to another gene. Although their study is very different than ours in the methodology followed (ChIP-chip *vs.* ChIP-seq) and the proteins targeted by the ChIP, comparing the two can be useful. Overall, we found the two datasets are comparable with respect to miRNA promoter characteristics. Furthermore, we ran the CPPP algorithm on their data and verified most of their predicted promoters. In particular, there are 44 TSSs associated with intergenic miRNAs with positive Marson score (after removing some inconsistent or mis-annotated TSSs, see *Materials and Methods*); and CPPP scored all but three of them as likely to contain a core promoter (see *Supplementary Data*). The two datasets (ours and Marson's) include one miRNA gene and one miRNA cluster with well studied TSSs: cluster miR-23a∼miR-27a∼miR-24-2 [5], and gene miR-21 [26]. CPPP correctly identified the location of both known TSSs, while the Marson dataset only found the correct TSS for the miR-23a cluster (see *Supplementary Data*.)

### Conclusions

The prediction of miRNA TSSs and the understanding of the processes that affect their transcription is an essential step towards deciphering their role in regulatory networks. In this study, high-throughput Pol II ChIP-chip data were collected and used to infer miRNA TSSs. Analysis of these data showed that intergenic and some intragenic miRNAs are transcribed by Pol II at a distance that can be as large as 40 Kb from the pre-miRNA genes, indicating that pri-miRNA transcripts might be much longer than originally thought [10], [11], [12]. We compared the most commonly used promoter features (*n*-mer frequencies and PSSM models) and found the *n*-mer frequencies to be generally better than the generalized PSSM models, at the cost of additional parameters. Also, in agreement with other studies [18], we found that CpG+ promoters are much easier to predict than CpG− and that core promoter prediction is more efficient when both models are used independently. However, we also found that this generally superior performance of the SVM models on CpG promoters *vs.* non-CpG promoters is due to the GC bias of the former. When a random background with similar GC content as the CpG+ promoters was used, the efficiency of the SVM model became similar to that of the CpG− model. This is a previously overlooked feature of the SVM training for core promoter recognition. Using the best performing SVM on our ChIP-chip data, we found that miRNA Pol II promoters contain most of the same features as the protein coding gene promoters.

Our results highlight the complexity and independence of the miRNA gene expression regulation and thus encourage more detailed studies in various cells, organs and physiological conditions. Our study gives a biochemical verification to previous statistical analyses that indicated that pri-miRNA transcripts can be tens of thousands of bases long [23]. Finally, the finding that 26% or more of the intragenic miRNA genes may be transcribed by their own promoter also encourages much more detailed studies into their transcriptional regulation.

Elucidating the transcriptional networks that determine expression of miRNAs is critically important considering their important regulatory roles. miRNA location arrays may be useful tools in elucidating these networks

## Materials and Methods

### Chromatin Immunoprecipitation (ChIP-chip)

Approximately 10^8^ A549 cells (American Type Culture Collection, Manassas, VA) were grown in F12K medium (Invitrogen, Carlsbad, CA) with 2 mM L-glutamine and 10% fetal bovine serum. Cells were incubated at 37°C in a humidified chamber supplemented with 5% CO_2_. Once 80% confluent, cells were serum starved overnight. Proteins were cross-linked to the DNA using fresh formaldehyde solution (50 mM Hepes-KOH pH 7.5, 100 mM NaCl, 1 mM EDTA pH 8.0, 0.5 mM EGTA pH 8.0, 11% Formaldehyde) for 10 min at room temperature. The formaldehyde was quenched with 2.5 M glycine for 5 min at room temperature. Cells were washed twice in PBS and harvested using a silicone scraper. Cells were centrifuged at 1,350×g for 5 minutes at 4°C and the pellet washed twice with PBS. The pellet was resuspended in 5 ml of lysis buffer 1 (50 mM Hepes-KOH pH 7.5, 140 mM NaCl, 1 mM EDTA, 10% glycerol, 0.5% NP-40, 0.25% Triton X-100) and rocked at 4°C for 10 min. The cells were centrifuged at 1,350×g for 5 minutes at 4°C and the pellet resuspended in 5 ml of lysis buffer 2 (10 mM Tris-HCl, pH 8.0, 200 mM NaCl, 1 mM EDTA, 0.5 mM EGTA), rocked at room temperature for 10 min. The nuclei were pelleted by centrifuging at 1,350×g for 5 minutes at 4°C. The pellet was resuspended in 5 ml of lysis buffer 3 (10 mM Tris-HCl, pH 8.0, 100 mM NaCl, 1 mM EDTA, 0.5 mM EGTA, 0.1% Na-deoxycholate, 0.5% N-lauroylsarcosine). The cells were sonicated for 7 cycles of 30 seconds ON and 60 seconds OFF at a power 7 using a sonic dismembrator Model 100 (Fisher Scientific, Waltham, MA). The cells were centrifuged at 20,000×g for 10 minutes at 4°C and 50 µl of the supernatant was set aside as the whole cell extract (WCE). The rest of the supernatant was incubated overnight at 4°C with 100 µl of Dynal Protein G magnetic beads that had been pre-incubated with either 10 µg RNA polymerase II antibody (Abcam, Cambridge, MA) or 10 µg E2F-4 antibody (Santa Cruz Biotechnology, Santa Cruz, CA). The beads were washed 7 times in RIPA buffer (50 mM Hepes-KOH pH 7.6, 500 mM LiCl, 1 mM EDTA pH 8.0, 1% NP-40, 0.7% Na-deoxycholate) and once in Tris-EDTA containing 50 mM NaCl. Elution was done in elution buffer (50 mM Tris-HCl pH 8.0, 10 mM EDTA pH 8.0, 1% SDS) for 15 min at 65°C. Reversal of crosslinks of the immunoprecipitate (IP) and the WCE was done at 65°C overnight. Cellular RNA was digested with 0.2 mg/ml RNaseA (Invitrogen) at 37°C for 2 h followed by protein digestion with 0.2 mg/ml proteinase K (Invitrogen) at 55°C for 30 min. The DNA was purified by phenol∶chloroform∶isoamyl alcohol extraction and ethanol precipitation. Purified DNA was blunted using T4 DNA polymerase (New England Biolabs, Ipswich, MA) and ligated to 2 µM linkers using T4 DNA ligase (New England Biolabs). The IP and the WCE was amplified in two stages of PCR and purified by phenol∶chloroform∶isoamyl alcohol extraction and ethanol precipitation. 2 µg each of IP and WCE was labeled with Cy5-dUTP and Cy3-dUTP (Perkin Elmer, Waltham, MA) respectively. Labeling was carried out by random-primed Klenow-based extension using the CGH Labeling kit (Invitrogen). The samples were cleaned up using Invitrogen's CGH columns included in the kit. 5 µg each of IP and WCE were combined with cot-1 DNA and the 10× blocking agent and 2× hybridization buffer supplied in the Agilent Oligo aCGH/ChIP-on-chip Hybridization Kit (Agilent, Santa Clara, CA). Hybridization was carried out in Agilent's SureHyb chambers at 65°C for 40 h in the DNA Microarray Hybridization Oven (Agilent). The slides were washed using Oligo aCGH/ChIP-on-chip wash buffer 1 and 2 (Agilent) and scanned in the DNA microarray scanner (Agilent). The scanned images were processed using Agilent's Feature Extraction software version 9.5.3

### ChIP-seq data

Marson *et al.* [14] recently published a study where they combined different ChIP-seq datasets for multiple DNA binding proteins (or modifications of them) to unravel the transcriptional machinery of the miRNA genes in mouse and human cells. We analyzed their human dataset with our CPPP and compared their results with ours. Their original dataset contained TSSs associated with 101 intergenic miRNA genes or gene clusters. We excluded 19 of them from this analysis, because we found them to either overlap with promoters of protein coding genes (10 TSSs) or were located downstream of the corresponding miRNA gene (4 TSSs) or there were genes found between the miRNA gene and the TSS prediction (5 genes; see *Supplementary Data*.) We also converted the coordinates to the current version of the human genome (hg18) using the *liftOver* utility of the UCSC Genome Browser [52]. This caused the miRNA-TSS distances to change significantly (more than 50 bp) for 7 of the 101 genes.

### miRNA Location Array Design

The miRNA location array was custom-made by Agilent with AMADID (Agilent Microarray Design Identifier) 014119. The array is available on the 44 K design. The probes tile 100 Kb regions (∼200 bp spacing) surrounding each miRNA and only in non-repeat masked regions. The probes are 45–60-mers, Tm balanced and map to the Hg17 database. There are 41585 probes from ∼164 miRNA intervals. Control probes such as GD (gene desert), intensity controls (LACC) and some negative controls were also included.

### Analysis of ChIP-chip Data

Median normalization of the log_2_ values of the ratio of signal to mock (precipitated DNA without antibody) was performed across the three-ChIP-chip arrays followed by a mean centralization to 0. Regions of Pol II binding were identified by the ChIPOTle sliding window method [22]; a window size of 1 Kb was used with a step size of 50 bp. The window was reported as significant if the *p*-value was below 0.05 after adjustment by the conservative Bonferroni correction method for multiple testing. Overlapping significant windows were combined and the region with the lowest *p*-value was reported.

### Gene Coordinate and Sequence Collection

Pol II core promoters were extracted from two databases: Eukaryotic Promoter Database [48] and DBTSS [53]. Between the two databases there were 3,015 unique human TSSs (1,744 from Eukaryotic Promoter Database and 1,271 from DBTSS as originally identified by Zhou *et al* [12]). The core promoter regions were partitioned into 1,445 that contained CpG islands and 1,570 that did not according to the method and threshold used in Zhao *et al* [21]. For the training and testing of the various SVM models the area [−450, +50] surrounding the TSS was used as the positive dataset. An equal number of 500 bp genomic sequences, randomly selected from the intergenic regions of all chromosomes were used as the negative dataset for the CpG− model. A second set of sequences was collected as described by Zhao *et al.* [21] This dataset had GC content similar to the CpG island promoter dataset and was used as negative dataset for the CpG− model. Special care was given so that the randomly selected regions were not located within 3 Kb from the 5′ end of any annotated gene.

Genomic coordinates for all mRNA TSSs, mRNA introns and miRNA were collected from the UCSC table browser [24], [25]. Intragenic miRNAs were identified as those found within an intron, exon or UTR of a mRNA and transcribed in the same orientation. All other miRNAs were labeled as intergenic.
